# Efficacy and safety of Epimedium total flavonoids for primary osteoporosis: a systematic review and meta-analysis

**DOI:** 10.3389/fphar.2024.1505926

**Published:** 2024-11-18

**Authors:** Jinkun Li, Xudong Huang, Yifeng Yuan, Haixin Zhang, Hang Zhou, Wei Xiong, Yuyi Li, Zechen Zhang, Fengqing Qie, Yingdelong Mao, Bocheng Liang, Xiaolin Shi, Kang Liu

**Affiliations:** ^1^ The Second School of Clinical Medicine, Zhejiang Chinese Medical University, Hangzhou, Zhejiang, China; ^2^ The Second Affiliated Hospital of Zhejiang Chinese Medical University (Xinhua Hospital of Zhejiang Province), Hangzhou, Zhejiang, China; ^3^ School of Physical Education and Health, Gansu University of Chinese Medicine, Lanzhou, Gansu, China; ^4^ Tokyo Yuxin Health Institute, Tokyo, Japan

**Keywords:** primary osteoporosis, Epimedium total flavonoids, traditional Chinese medicine, bone mineral density, bone metabolism

## Abstract

**Background:**

Epimedium total flavonoids (EF) have been recommended to be one of the effective components in Traditional Chinese Medicine (TCM) for the treatment of primary osteoporosis (POP) in China. Due to the lack of evidence-based medical evidence on the efficacy and safety of EF for the treatment of POP, the current systematic review and meta-analysis was carried out aimed at evaluating the curative effects and safety profile of EF treatment for POP in order to provide decision making references for clinical research.

**Methods:**

The PubMed, Cochrane Library, EMBASE, Web of Science, CNKI, Wanfang, and VIP databases were searched from the date of inception to 11 August 2024. The outcomes of effectiveness and safety of included studies were collected to conduct meta-analysis or systematic review.

**Results:**

A total of 6 RCTs were included in this study, involving 838 participants. Overall, our results revealed that the experimental group (EG) had comparable results of efficacy to the control group (CG). The lumbar vertebra bone mineral density (BMD) was statistically different compared to the CG (MD = 0.03; 95% CI: 0.01, 0.04; *p* = 0.0003), but the clinical significance deserved consideration because the 95% CI nearly crossed the invalid line. The femoral neck BMD was neither statistically different nor clinically different between two groups (MD = 0.00; 95% CI: 0.01, 0.02; *p* = 0.67). The total complication rates were comparable among the two groups (RR = 0.68; 95% CI: 0.39, 1,19; *p* = 0.18). The quality of the evidence of the present study was judged as moderate and low based on the GRADE analysis.

**Conclusion:**

EF treatment exhibited good curative effects and safety. The result was comparable to the CG, including other Chinese patent medicines and calcium and vitamin D supplements. The EF treatment was proved to be a reliable alternative option for POP.

## 1 Introduction

Osteoporosis (OP) is a condition characterized by a decrease in bone density and the deterioration of bone tissue, which leads to increased bone fragility and a higher risk of fractures (Jiang et al., 2024). It commonly affects older adults, particularly postmenopausal women, due to decreased estrogen levels which accelerate bone loss. Symptoms of OP may include back pain, a loss of height, and frequent fractures, often occurring in the spine, hip, or wrist with minimal or no trauma (Camacho et al., 2020; Li et al., 2024). In recent years, the incidence of OP has gradually increased with high rates of multiple complications and mortality (ACOG, 2022).

OP is classified into primary and secondary OP according to the etiology. Typically, primary OP (POP), including postmenopausal osteoporosis (PMOP) and senile osteoporosis (SOP), is treated with some medications such as bisphosphonates (BPs), denosumab, selective estrogen receptor modulators (SERMs) and teriparatide (a kind of Parathyroid Hormone Analogs) (Meng et al., 2024). Early detection and appropriate treatment can help manage OP effectively and reduce the risk of fractures and mortality. However, the long-term safety and efficacy of these medications remain a concern, and issues such as potential side effects, including atypical femoral fractures and osteonecrosis of the jaw, continue to be debated (Wei et al., 2024). There is a long history of using traditional Chinese medicine (TCM) for the treatment of various diseases. In recent years, TCM has become an effective alternative for the treatment of OP, and some TCM treatments and active ingredients have become popular with doctors and patients due to their ease of use, therapeutic effect comparable to Western medicine, and minimal side effects (Wei et al., 2024; Zhou et al., 2024).

According to the relevant guidelines, Epimedium total flavonoids (EF) is one of the effective components in TCM of which the drug efficacy is relatively clear (Zhenlin, 2023). Many excellent studies reported some unexpected therapeutic effects of some effective components on some certain diseases (Ullah et al., 2024). Modern pharmacological experiments have shown that the main ingredients of EF include icaritin, Baohuoside and epimedin. Large number of basic research have shown that the EF could improve the osteogenic capability in the animal and cell model. It was demonstrated that the EF could regulate bone metabolism, increase bone mass in osteoporotic rats by up-regulating the expression of TGF-β1 and Smad2 (Xiao et al., 2022). Other studies showed that the EF could inhibit the apoptosis of osteoblasts to promote the bone formation and inhibit osteoclast generation to decrease the bone absorption (Xia et al., 2023). There was a study reported that the EF could ameliorate osteoporosis in ovariectomized rats by targeting Cullin 3/Nrf2/OH pathway for osteoclast inhibition (Si et al., 2024). Another study found that the EF could ameliorate POP by targeting AKT/mTOR/ULK1 autophagy signaling pathway (Zou et al., 2024). By the way, other authors reported in their study that EF could promote the proliferation and osteogenic differentiation of bone-derived mesenchymal stem cells in patients with osteoporosis and T2DM by upregulating GLI-1 (Xia et al., 2023). Moreover, the EF capsule, a kind of Chinese patent medicine of which the EF is the major active ingredient, has already been approved for clinical use by the National Medical Products Administration (NMPA) of the People’s Republic of China and listed on the market (approval number: national drug standard Z20140012).

Although there are many basic research studies on EF on POP treatment, there is still a dearth of relevant evidence-based medical data on EF efficacy and safety. Therefore, we conducted a systematic review and meta-analysis on the efficacy and safety of EF in POP treatment, providing a reference for the appropriate application of EF in clinical practice.

## 2 Methods

This research was registered with the international prospective register of systematic reviews (PROSPERO number: CRD42024550573). This work has been reported in line with PRISMA (Preferred Reporting Items for Systematic Reviews and Meta-Analyses).

### 2.1 Search strategy

We identified the relevant studies by conducting a comprehensive literature search of the PubMed, Cochrane Library, EMBASE, Web of Science, CNKI, Wanfang and VIP databases from the date of inception to 11 August 2024. A combination of subject words and free words were used for searching. The keywords included: “Epimedium total flavonoid”, “Epimedium flavonoid”, “icariin”, “icaritin”, “Baohuoside”, “Chao-huo-ding”, “epimedin”, “Osteoporosis, Postmenopausal” and “Senile Osteoporosis”. The detailed searching process is presented in the Supplementary Material. No study types restriction was applied when searching literature. Moreover, we manually searched the reference lists of previously published reviews and included studies for additional pertinent trials.

### 2.2 Inclusion and exclusion criteria

The inclusion criteria were as follows: (1) patients definitively diagnosed with POP including PMOP, SOP, and osteoporotic fractures in POP; (2) patients in the experimental group (EG) given EF (including various forms of flavonoids extracted from Epimedium) in combination or not with other treatments, while patients in the control group (CG) given any type of treatment; (3) trials with human studies; (4) it is possible to synthesize all or any of the trial outcome data.

Studies were excluded if: (1) published data were insufficient and corresponding authors did not respond the request for further information; (2) single-arm studies that only include one group; (3) conference abstracts, experimental studies and basic studies.

### 2.3 Study selection

The eligibility of the title and abstract of the references obtained during the literature searching was evaluated by two independent reviewers. Consensus was used to resolve any disagreements. After that, full text publications were screened for possibly qualifying studies, and any discrepancies were settled by consensus. Whenever necessary, the third author made decisions.

### 2.4 Data extraction

A standardized data extraction form was used to extract the following information: Authors, publication year, country, study design, sample size, subject age, duration of follow-up, clinical outcomes and other relevant data. The outcomes of each included study were extracted to conduct meta-analyses. Other relevant outcomes that were not enough for meta-analyses were described as part of the systematic review. The data extraction will be carried out independently by two researchers. If differences between the two researchers are found, a third researcher will examine the data that has been retrieved and address them.

### 2.5 Assessment of risk of bias

Two reviewers independently evaluated the included studies’ methodological quality and bias risk. The Cochrane risk-of-bias criteria was used for the randomized controlled trial (RCT) (Higgins and Green, 2008). The following 7 aspects were assessed: randomization sequence generation, allocation concealment, blinding of participants and personnel, blinding of outcome assessment, incomplete outcome data, selective reporting and other bias. Studies sponsored by implant firms and those in which the baseline characteristics of the various intervention groups were not comparable were considered to have other bias. According to Zhao’s standards, the methodological quality of RCTs were rated as low quality, high quality, or moderate quality (Zhao et al., 2017).

Assessment of the methodological quality of case-controlled studies and cohort studies was conducted using the Newcastle-Ottawa quality assessment scale (NOS) (Wells, 2014). The three items were evaluated: selection, comparability, and exposure. Each trail was assigned a score of 0–9. The trials scored ≥7 were considered to be of high quality.

As for the non-randomized controlled trials, the Methodological Index for Non-randomized Studies (MINORS) was used to assess the methodological quality (Slim et al., 2003). Trials with MINORS scores ≤12 were considered low quality.

### 2.6 Data synthesis

The data was synthesized used the Cochrane Review Manager (RevMan) software version 5.4 (The Nordic Cochrane Center, The Cochrane Collaboration Copenhagen, Denmark). As for continuous data, inverse variance method was used to calculate the mean differences (MD) with a 95% confidence interval (CI) assessing the effect size. While for dichotomous variables, Mantel-Haenszel analysis method was utilized to calculate the risk ratio (RR) with a 95% CI assessing the effect size. The heterogeneity among the included studies was assessed using the I^2^ statistic. The random effect model will be used when the value of I^2^ is greater than 50%; otherwise, the fixed effect model will be used.

### 2.7 Sensitivity analysis

Sensitivity analysis was carried out to address possible heterogeneity and determine the robustness of the results. It was conducted by excluding each literature by turn that would increase the heterogeneity of each outcome in order to ascertain whether particular characteristics would change the overall effects of each outcome (Wang et al., 2018). In the present study, the sensitivity analysis was conducted in the comparison which included at least 3 articles.

### 2.8 Assessment of quality of the evidence

Two authors independently evaluated the overall quality of the evidence based on the guidelines of the Grading of Recommendations Assessment, Development and Evaluation (GRADE) (Grade Working Group, 2004) using the online software GRADE pro GDT (https://www.gradepro.org/). The quality of the evidence was classified as high, moderate, low or very low.

## 3 Results

### 3.1 Literature search and baseline characteristics

**FIGURE 1:**
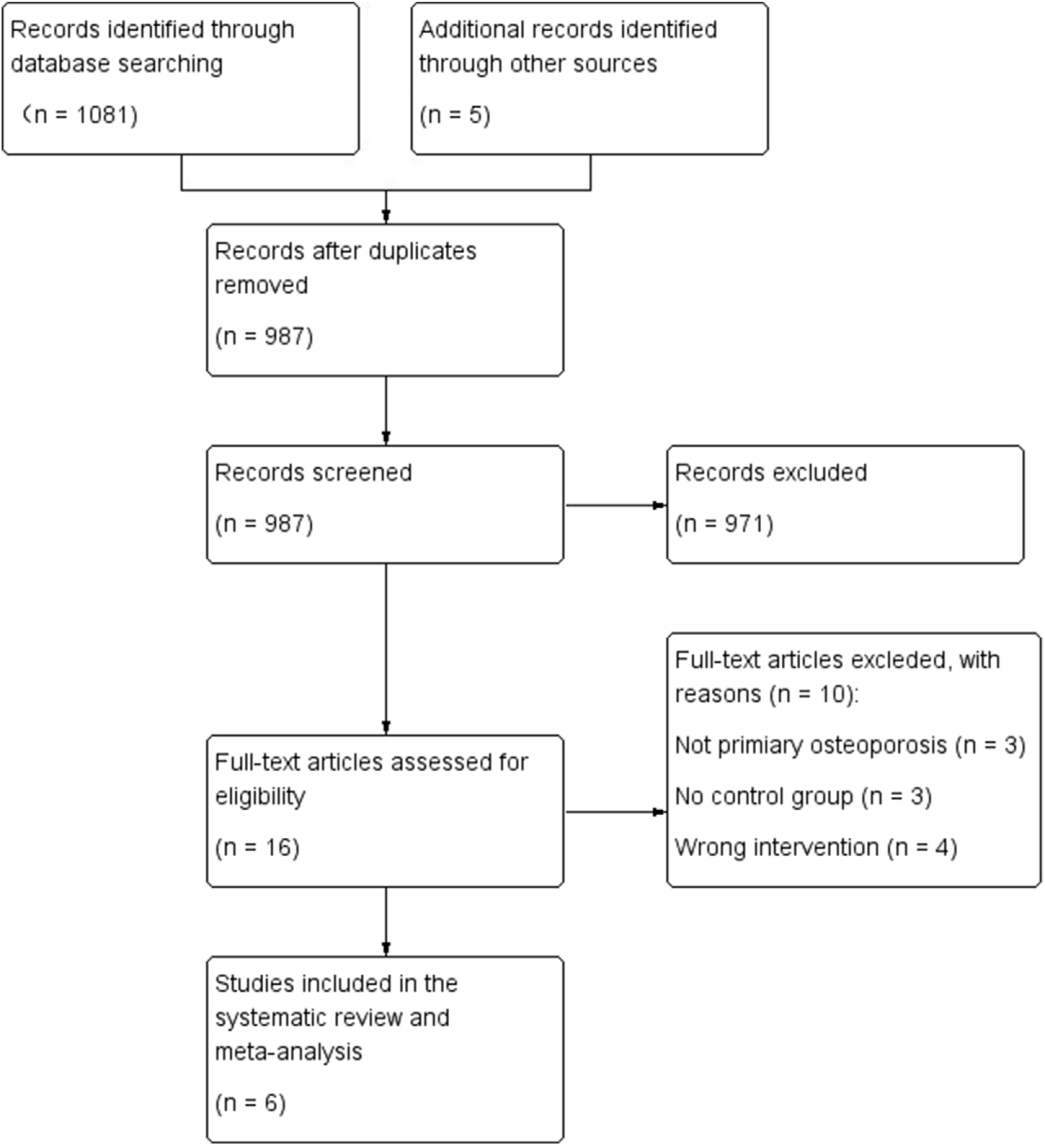
Flow diagram of literature searching and selection process.

The process of literature selection is shown in Figure 1. We identified a total of 1,081 initial references, and an additional 5 papers through other sources. 16 studies with full text remained to be assessed for eligibility. Out of these 16 references, 6 RCTs were included (Zhang et al., 2007; Shou et al., 2009; Lu et al., 2013; Jin, 2019; Xingfa and Jiang, 2020; Yong et al., 2021). Other studies were excluded for various reasons (Figure 1).

The summary characteristics at baseline of included studies are presented in the Table 1. All 6 studies reported the comparable demographic data at baseline. A total of 838 subjects were included in the 6 studies. Other details are described in the Table 1. Moreover, all the medication methods reported in the included literature were oral capsules. However, specific drug preparation processes are not known.

**TABLE 1:** Baseline characteristics of the included studies.

Studies	Country /Region	Study design	Sample size EG/CG	Gender (M/F)	Mean age (EG/CG, y)	Intervention (EG/CG)	Follow-up (months)	Outcome
Jin (2019)	China	RCT	26/26	0/52	69.27 ± 5.46/70.13 ± 6.85	EF capsules + CG/Calcium Carbonate D3 Tablets	2, 6	①②③
Lu et al. (2013)	China	RCT	360/120	54/426	61 (50-77)/63 (49-75)	EF capsules/Gusongbao caosules	6	①③
Shou et al. (2009)	China	RCT	32/32	11/53	62 ± 6/62 ± 7	EF capsules/Gushukang capsules	6	①④⑦
Xingfa and Jiang (2020)	China	RCT	42/42	32/52	72.13 ± 6.94/71.91 ± 7.05	EF capsules + CG/Calcium Carbonate D3 Tablets + Alfacalcidol	6	①③
Yong et al. (2021)	Singapore	RCT	29/29	0/58	56.9 ± 11.8/57.0 ± 11.6	Epimedium prenylflavonoids/placebo	0.75, 1.5, 2	②③④⑥
Zhang et al. (2007)	Hong Kong	RCT	50/50	0/100	64 ± 4/63 ± 3	Epimedium-Derived Phytoestrogen Flavonoids/placebo	12, 24	①⑤

Abbreviations: EG, experimental group; CG, control group; M, male; F, female; RCT, randomized controlled trial; EF, Epimedium total flavonoids. ① = bone mineral density (BMD); ② = serum procollagen type I N-propeptide (P1NP) and C-terminal telopeptide of type I collagen (CTX) levels; ③ = complications; ④ = serum bone specific alkaline phosphatase (BALP) levels; ⑤ = serum estradiol (E2), serum osteocalcin (OC), urine deoxypyridinoline (DPD) and endometrial thickness; ⑥ = levels of the tumor necrosis factor receptor associated factor 6 (TRAF6); ⑦ = the levels of serum calcium and phosphorus.

### 3.2 Risk of bias assessments in the included studies

**FIGURE 2:**
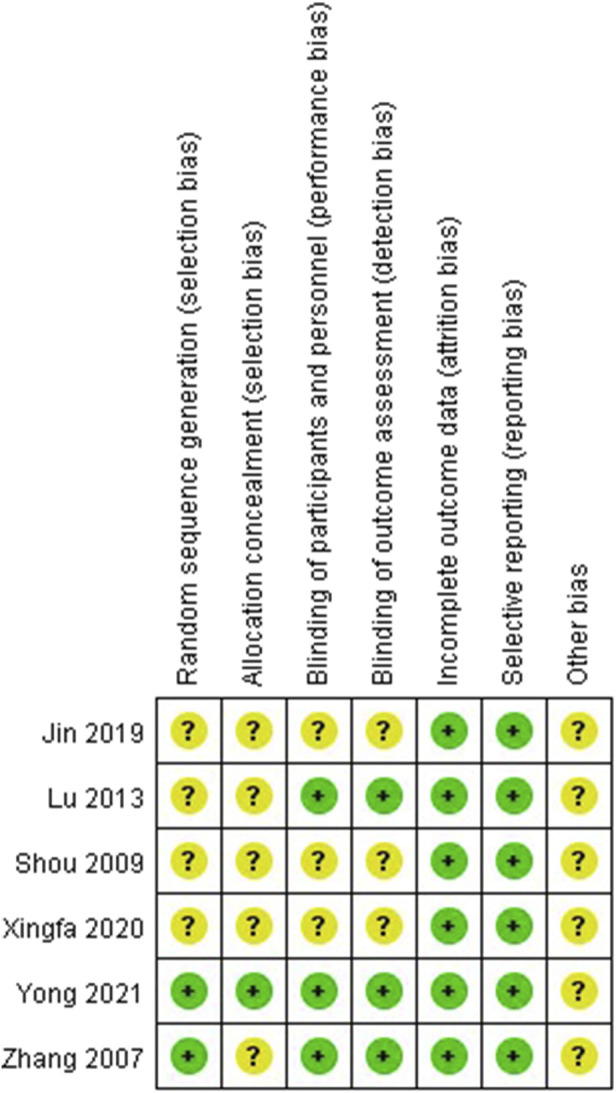
The methodological quality evaluation of randomized controlled trials.

The risk of bias assessments was presented in Figure 2. All studies were at low risk of bias regarding incomplete outcome data and selective reporting. Only two RCTs (Zhang et al., 2007; Yong et al., 2021) were at low risk of bias regarding random sequence generation and only one RCT (Yong et al., 2021) was at low risk of bias regarding allocation concealment. According to the Zhao’s criteria, only Yong’s research was able to be considered high quality while others were considered to moderate quality (Zhao et al., 2017).

### 3.3 Outcome measurements

In the included studies, 5 studies evaluated the bone mineral density (BMD) as one of the indicators of clinical outcomes (Zhang et al., 2007; Shou et al., 2009; Lu et al., 2013; Jin, 2019; Xingfa and Jiang, 2020). Two studies reported the serum procollagen type I N-propeptide (P1NP) and C-terminal telopeptide of type I collagen (CTX) levels (Jin, 2019; Yong et al., 2021). Two studies reported the serum bone specific alkaline phosphatase (BALP) levels (Shou et al., 2009; Yong et al., 2021). Four studies reported the complications (Lu et al., 2013; Jin, 2019; Xingfa and Jiang, 2020; Yong et al., 2021). A few other results that were insufficient for meta-analysis were also described, which were also documented as a part of systematic review.

#### 3.3.1 Effect of epimedium total flavonoids on BMD

##### 3.3.1.1 Lumbar vertebra BMD

**FIGURE 3:**
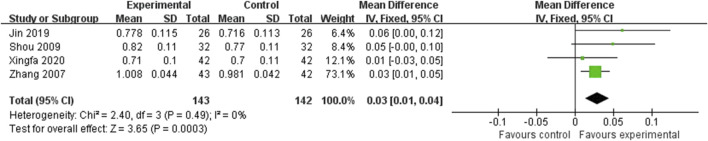
Forest plot of the lumbar vertebra BMD. There was a statistically significant difference between the two groups.

Four studies reported the lumbar vertebra BMD, involving 285 subjects (Zhang et al., 2007; Shou et al., 2009; Jin, 2019; Xingfa and Jiang, 2020). The result of this comparison was presented in Figure 3. The values of lumbar vertebra BMD were statistically different between two groups (MD = 0.03; 95%CI: 0.01, 0.04; P = 0.0003) without heterogeneity (I^2^ = 0%, P = 0.49). From the Figure 3, the total 95%CI nearly crosses the invalid line. Therefore, even though the result was statistically significant, such small differences might not have clinical significance.

##### 3.3.1.2 Femoral neck BMD

**FIGURE 4:**
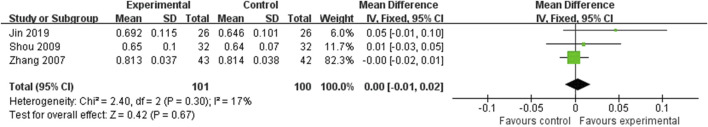
Forest plot of the femoral neck BMD. There was no statistically significant difference between the two groups.

Three studies reported the femoral neck BMD, involving 201 subjects (Zhang et al., 2007; Shou et al., 2009; Jin, 2019). The result of this comparison was presented in Figure 4. The values of femoral neck BMD were not statistically different between two groups (MD = 0.00; 95%CI: 0.01, 0.02; P = 0.67) with low heterogeneity (I^2^ = 17%, P = 0.30). From the Figure 4, the total 95%CI crosses the invalid line. Therefore, the result was neither statistically different nor clinically different.

##### 3.3.1.3 Other sites BMD and the rate of change in BMD

There was one RCT that reported the Ward’s triangle BMD, greater trochanter of femur BMD and hip BMD (Shou et al., 2009) and one RCT that reported the rate of change in BMD (Lu et al., 2013). In Shou’s study, he reported that the values of BMD in the EG and CG was 0.47 ± 0.11 vs. 0.45 ± 0.09, 0.51 ± 0.10 vs. 0.51 ± 0.07, 0.71 ± 0.11 vs. 0.70 ± 0.08, respectively. Meanwhile, the differences between two groups were not statistically significant (P > 0.05).

In Lu’s study, the author presented the rate of change in BMD as the outcome [(BMD after treatment–BMD before treatment)/BMD before treatment × 100%]. The author defined the “markedly effective” as the rate of change in BMD was greater than or equal to the least significant change (LSC), “effective” as the rate was between the positive and negative LSC, and “invalid” as the rate was less than the negative value of LSC. The total effective rate in EG was higher than the rate in CG and the difference was statistically significant (P = 0.0367). However, Lu did not give enough specific values of BMD sufficient to extract numerical data to conduct meta-analysis.

#### 3.3.2 Serum P1NP and CTX levels

**FIGURE 5:**
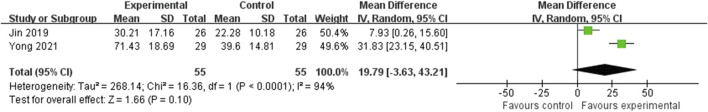
Forest plot of the serum P1NP levels. There was no statistically significant difference between the two groups.

**FIGURE 6:**
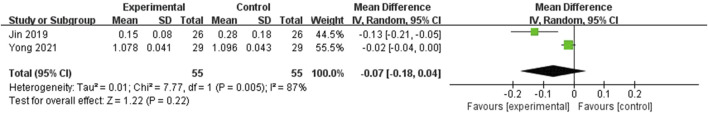
Forest plot of the serum CTX levels. There was no statistically significant difference between the two groups.

There were 2 RCTs that reported the serum P1NP and CTX levels involving 110 subjects (Jin, 2019; Yong et al., 2021). The results were shown in the Figures 5, 6. As for the serum P1NP level, the level in EG was significantly higher than the level in CG (MD = 18.41; 95%CI: 12.66, 24.16; P < 0.001), however, with the high heterogeneity (I^2^ = 94%, P < 0.001). About the serum CTX level, the result in EG was significantly better than the CG (MD = −0.03; 95%CI: 0.05, −0.01; P = 0.01), however, with the high heterogeneity (I^2^ = 87%, P = 0.005).

#### 3.3.3 Serum BALP levels

**FIGURE 7:**
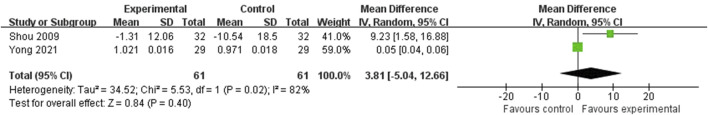
Forest plot of the serum BALP levels. There was no statistically significant difference between the two groups.

There were 2 RCTs that reported the serum BALP levels involving 122 subjects (Shou et al., 2009; Yong et al., 2021). Yong et al. reported the values that were pre-to post-intervention changes, but Shou et al. reported the values before and after intervention, respectively. According to the Cochrane Handbook (Higgins and Green, 2008), the latter was unified to the value of pre-to post-intervention change. As shown in the Figure 7, the difference was not statistically significant (MD = 3.81; 95%CI: 5.04, 12.66; P = 0.40) with the high heterogeneity (I^2^ = 82%, P = 0.02). Besides, the total 95%CI crosses the invalid line. Therefore, the difference of serum BALP levels between two groups was not considered to be clinically significant.

#### 3.3.4 Complications

**FIGURE 8:**
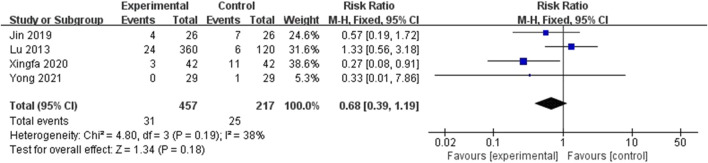
Forest plot of the complications. There was no statistically significant difference between the two groups.

Out of 6 included studies, only 4 studies were included in meta-analysis regarding total complications (Lu et al., 2013; Jin, 2019; Xingfa and Jiang, 2020; Yong et al., 2021). According to the Figure 8, the total complication rates was comparable among the two groups (RR = 0.68; 95%CI: 0.39, 1,19; P = 0.18) and the heterogeneity existed (I^2^ = 38%, P = 0.19). The total 95%CI crosses the invalid line. Therefore, conclusions about the reduction or increasing role of EF in total complications could not be obtained.

However, authors did not report the number of patients with specific complications in all 4 studies. The reported complications included skin rash, constipation, diarrhea, palpitation, aphtha and xerostomia. Beyond these complications, Yong et al. (Yong et al., 2021) reported the changes of levels of some biochemical markers, including hepatic markers (AST, ALP, LDH), hematological markers (Hb) and renal markers (Urea). Based on the description in the literature, all these changes reverted to normal subsequently.

#### 3.3.5 Some other outcomes

Some other results that were insufficient for meta-analysis were also reported. Zhang et al. reported serum estradiol (E2), serum osteocalcin (OC), urine deoxypyridinoline (DPD) and endometrial thickness (Zhang et al., 2007). For bone resorption markers, a significantly different pattern of urine DPD change was found between the EG and the CG (P = 0.002 for interaction between time and group). Urine DPD in the CG did not change significantly from baseline at 12 and 24 months (P > 0.05), whereas urine DPD in the EG was significantly decreased 43% from baseline at 12 months and 39% at 24 months (P < 0.05). A significant difference in DPD between the EG and the CG was found at 12 and 24 months (P < 0.05). For bone formation markers, serum OC in the CG decreased 3.9% from baseline to 12 months and down 6.9% at 24 months (P > 0.05). However, serum OC in the EG increased 5.6% from baseline to 12 months and up 10.7% at 24 months with no statistical significance (P > 0.05). Besides, no significantly different change in OC was found at 12 or 24 months (P > 0.05). Similarly, changes in serum E2 and endometrial thickness at 12 and 24 months were also not significantly different from baseline (P > 0.05).

Yong et al. evaluated the tumor necrosis factor receptor associated factor 6 (TRAF6) protein in osteoclast-precursor monocytes in peripheral blood (Yong et al., 2021). There was a trend towards lower levels of TRAF6 in peripheral blood monocytes with the extension of time, but this trend was not reaching statistical significance (P > 0.05).

Shou et al. measured the levels of serum calcium and phosphorus before and after treatment (Shou et al., 2009). The levels of serum calcium and phosphorus before and after treatment in EG were 2.35 ± 0.10 vs. 2.42 ± 0.13 and 1.19 ± 0.18 vs. 1.24 ± 0.20. In CG, the levels were 2.33 ± 0.12 vs. 2.41 ± 0.09 and 1.24 ± 0.19 vs. 1.29 ± 0.26. The levels of serum calcium after treatment in both two groups were significantly higher than the levels before treatment (P < 0.05). However, the authors did not state whether the difference was statistically significant in other comparisons.

### 3.4 Sensitivity analysis

As mentioned, sensitivity analysis was carried out for lumbar vertebra BMD, femoral neck BMD, and the total complications. The outcome demonstrated that leaving out any single study for each comparison had no bearing on the total effect.

### 3.5 GRADE analysis for the quality of the evidence

**FIGURE 9:**
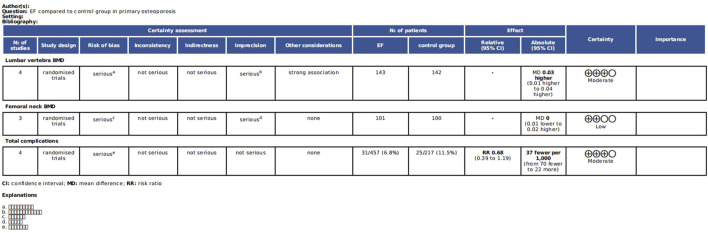
The quality of the evidence of lumbar vertebra BMD, femoral neck BMD and total complications based on the GRADE analysis.

Only the comparisons that included at least 3 studies were ultimately available for GRADE analysis. According to our results, the quality of the evidence of lumbar vertebra BMD, femoral neck BMD, and total complications was analyzed by GRADE system. The quality of the evidence was judged as moderate and low (Figure 9). This is mainly because of the relatively small sample size and the unclear risk of bias in the aspect of random sequence and blinding. See Figure 9 for more details.

## 4 Discussion

As the world’s population ages, the incident rate of POP is increasing globally (Chen et al., 2024). POP, because of its tendency to lead to osteoporotic fractures, is a major risk factor for disability and even death of older patients and can significantly impair the quality of life (Shen et al., 2024). POP has been a major worldwide public health problem. At present, interventions for POP include inhibiting bone resorption and promoting bone formation. However, treatment with western medications alone remains controversial. TCM has shown effective in treating POP. Many TCM compounds used to treat POP contain Epimedium as a major herb (Shou et al., 2009). EF, as one of the effective ingredients of Epimedium, has been described to display anti-osteoporosis effects by lots of basic research. Meanwhile, EF capsules are already on the market for POP. The aim of this systematic review and meta-analysis was to evaluate the clinical efficacy and safety of EF in the treatment of POP and serve clinical evidence.

In this study, 6 outcomes were compared including lumbar vertebra BMD, femoral neck BMD, serum P1NP levels, serum CTX levels, serum BALP levels and total complications in the meta-analysis. At the same time, some other outcomes which were insufficient to conduct meta-analysis were also reported as the part of systematic review. As for the BMD, the outcome of meta-analysis showed that the lumber vertebra BMD in EG was significantly higher than CG (P < 0.05) with no heterogeneity. But the pooled 95%CI nearly crossed the invalid line (Figure 3). Therefore, although the statistical difference was meaningful, the clinical difference between two groups was considered small. Moreover, about the femoral neck BMD, the difference was neither statistically nor clinically meaningful (Figure 4). Our results were similar to other studies. In Shou’s study, the authors described the Ward’s triangle BMD, greater trochanter of femur BMD and hip BMD after EF treatment, and the result showed that there was no significant difference between EG and CG (Shou et al., 2009). Another study reported the significant difference between EG and CG about the rate of change in BMD (Lu et al., 2013). The authors observed that the outcome in the EG was significantly better than in the CG, but they did not describe specific values of BMD. Many basic studies have reported that the EF could improve the proliferation and osteogenic differentiation of osteoblasts and inhibit the activity and osteoclast differentiation of osteoclasts (Si et al., 2024; Yang et al., 2024; Zhang et al., 2024). Our data exhibited that there was no superior efficacy compared to the CG, including other Chinese patent medicines, Calcium and vitamin D supplements. The EF gained the comparable efficacy compared with these medicines. Bone reconstruction is a dynamic and complicated process, and the change of the BMD is influenced by many factors. Therefore, we thought that an idealized increase in BMD is difficult to achieve.

About the serum biomarkers, we compared the levels of P1NP, CTX and BALP. P1NP and BALP were typical biomarkers of osteogenic ability, CTX was typical biomarkers of osteoclastic abilities. We observed that the levels of P1NP and BALP in EG was higher than CG and the level CTX in EG was lower than CG. Our observations are similar to the previous study (Zhang et al., 2021; Zou et al., 2024). However, the results of this study also showed that there was no statistically significant difference between two groups among these three comparisons. Besides, the pooled 95%CI crossed the invalid line among these three comparisons (Figures 5–7). Thus, similar to the BMD, the effect of EF was comparable to the treatment in CG among the levels of serum biomarkers. However, the heterogeneity in these comparisons were relatively high. This might be because of that there were only two studies included in these comparisons causing the sensitive analysis was not able to be conducted. We have used the random effects model to conduct meta-analysis to minimize the heterogeneity.

About the safety of the treatment of EF, we evaluated the total complications in the meta-analysis. Our results revealed that the rate of total complications in EG was lower than CG but the difference between two groups was not statistical and the pooled 95%CI crossed the invalid line (Figure 8). No serious specific complications were reported in all studies. This is similar to the findings of Yong et al. and others (Jin, 2019; Yong et al., 2021). It is worth noting that in Yong’s study, the authors tested the levels of some biochemical parameters including hepatic markers, hematological markers and renal markers. The author did find some levels outside the normal range, but all these changes reverted to normal subsequently and no substantial adverse clinical consequences was observed. Given this, the EF has a relatively good safety profile.

Some other outcomes that were insufficient for meta-analysis were also documented. These outcomes included bone formation marker (OC), bone absorption markers (DPD and TRAF6) and general markers (E2, serum calcium and phosphorus). In general, the EF could decrease bone absorption markers and increase the bone formation marker. However, most of these differences were not significant (Zhang et al., 2007; Shou et al., 2009; Yong et al., 2021).

There are some limitations in the present study. Firstly, the number of studies that met the inclusion criteria was relatively small, causing the inability to conduct a more comprehensive meta-analysis. Secondly, the number of high-quality studies was small, and the quality of the evidence was low and moderate based on the GRADE analysis, which may affect the credibility of the results of our meta-analysis. Thirdly, evaluating the long-term effect and safety of the EF requires extended follow-up data. Fourthly, the studies were limited to the country or region a country whose majority population is ethnic Chinese, and publication bias might be introduced, but it was not assessed in the present study because of the small number of included studies. Fifthly, common therapeutic drugs for treatment of POP like BPs were not presented in the included studies. Meanwhile, this article has some advantages. Our results revealed that EF could achieve good clinical effects and safety in terms of improving BMD and reducing complications. As far as we know, the present study was the first study to conduct a meta-analysis about the efficacy and safety of EF on POP, which might fill the relevant knowledge gap and provide the clinical guidance and evidence.

## 5 Conclusion

The present systematic review and meta-analysis revealed that the EF treatment for POP could achieve good curative effects and safety. Based on the findings of this study, the conclusion cannot be drawn that the EF exhibit better efficacy and safety compared with other Chinese patent medicines, Calcium and vitamin D supplements. The safety profile of the EF treatment was good which was comparable to other medications addressed above. The EF treatment was proved to be a reliable alternative option for POP. Regrettably, no RCT with high quality was included in our study and the quality of evidence provided by the present study was not assessed as high. In the future, more well-designed clinical trials with a large participant pool and extended follow-up time are needed to increase the quality of evidence and synthesize more data.

## Data Availability

The raw data supporting the conclusions of this article will be made available by the authors, without undue reservation.
