# Supplementary material for: Efficacy and safety of Epimedium total flavonoids for primary osteoporosis: a systematic review and meta-analysis
Source: Front Pharmacol. 2024 Nov 18;15:1505926. doi: 10.3389/fphar.2024.1505926 (PMC11608984; doi:10.3389/fphar.2024.1505926)
Supplement: Supplementary file 2 [file Table1.DOC]

Search Name:

Date Run: 11/08/2024 19:00:31

Comment:

ID Search Hits

#1 (Epimedium flavonoid):ti,ab,kw OR (Epimedium total flavonoid):ti,ab,kw (Word variations have been searched) 7

#2 MeSH descriptor: [Epimedium] explode all trees 10

#3 (Epimedium):ti,ab,kw OR (Epimedium grandiflorum):ti,ab,kw OR (Epimedium sagittatum):ti,ab,kw (Word variations have been searched) 50

#4 #2 OR #3 50

#5 MeSH descriptor: [Epimedium] explode all trees 10

#6 ("bioflavonoid"):ti,ab,kw OR ("flavonoid"):ti,ab,kw (Word variations have been searched) 2020

#7 #5 OR #6 2027

#8 #4 AND #7 14

#9 #1 OR #8 14

#10 MeSH descriptor: [Osteoporosis, Postmenopausal] explode all trees 2657

#11 ("post-menopausal osteoporoses"):ti,ab,kw OR ("post-menopausal osteoporosis"):ti,ab,kw OR ("postmenopausal osteoporosis"):ti,ab,kw OR ("postmenopausal osteoporoses"):ti,ab,kw OR (Perimenopausal Bone Loss):ti,ab,kw (Word variations have been searched) 2098

#12 ("postmenopausal bone loss"):ti,ab,kw OR ("perimenopausal bone loss"):ti,ab,kw OR (Osteoporosis, Postmenopausal):ti,ab,kw OR (Osteoporosis, Postmenopausal):ti,ab,kw (Word variations have been searched) 5696

#13 #10 OR #11 OR #12 5745

#14 MeSH descriptor: [Osteoporosis] explode all trees 5509

#15 (Age-Related Osteoporosis):ti,ab,kw OR (Age-Related Osteoporoses):ti,ab,kw OR (Age Related Osteoporosis):ti,ab,kw OR (Age-Related Bone Loss):ti,ab,kw OR (Senile Osteoporoses):ti,ab,kw (Word variations have been searched) 2249

#16 (Senile Osteoporosis):ti,ab,kw OR (Osteoporosis, Involutional):ti,ab,kw OR (Osteoporosis):ti,ab,kw (Word variations have been searched) 12503

#17 #14 OR #15 OR #16 12579

#18 #13 OR #17 12661

#19 #9 AND #17 6
